# Behavior of Shiga Toxin-Producing *Escherichia coli* in Naturally Contaminated Bovine Raw Milk Cheese During Aging

**DOI:** 10.3390/foods15173102

**Published:** 2026-09-01

**Authors:** Giulia Weiss, Ilaria Prandi, Sonia Rodas, Marzia Mancin, Andrea Massaro, Nicola Cologna, Marco Nardelli, Marco Ramelli, Barbara Cardazzo, Simone Belluco, Rosaria Lucchini

**Affiliations:** 1 Istituto Zooprofilattico Sperimentale delle Venezie, 35020 Legnaro, Italy; gweiss@izsvenezie.it (G.W.); srodas@izsvenezie.it (S.R.); mmancin@izsvenezie.it (M.M.); amassaro@izsvenezie.it (A.M.); sbelluco@izsvenezie.it (S.B.); rlucchini@izsvenezie.it (R.L.); 2 Trentingrana Consorzio dei Caseifici Sociali Trentini, 38121 Trento, Italy; cologna@concast.tn.it (N.C.); nardelli@concast.tn.it (M.N.);; 3 Department of Comparative Biomedicine and Food Science, University of Padua, 35020 Legnaro, Italy; barbara.cardazzo@unipd.it

**Keywords:** STEC, washed-rind cheese, microbial distribution, sampling methodology, cheese aging, own-check

## Abstract

Shiga toxin-producing *Escherichia coli* (STEC) are foodborne pathogens of concern in raw milk dairy products. Ensuring product safety requires regular own-check testing, but evidence on naturally occurring STEC behavior during aging remains limited. This study analyzed three batches of bovine raw milk washed-rind cheese produced with STEC-positive curds to evaluate: (I) STEC distribution within cheese wheels and (II) STEC behavior during aging. STEC detection followed ISO/TS 13136:2012. First, two cheese wheels from batch A were sampled at the core (n = 4) and external (n = 4) locations at three timepoints (55, 85, and 150 days after production). External regions showed higher detection rates than the core, but this pattern was inconsistent, indicating heterogeneous STEC distribution. Second, batches B (n = 25) and C (n = 24) were sampled at four timepoints (85–86, 121–122, 170–171, and 197–198 days after production). In batch B, 16/25 wheels tested positive at least once, with alternating positive/negative patterns. In batch C, no wheel remained consistently negative, and only 2/24 wheels were negative at the final timepoint. These findings highlight the heterogeneous distribution and prolonged detectability of STEC in semi-cooked bovine raw milk cheese up to 198 days of aging, emphasizing the challenges associated with STEC sampling and detection in this product.

## 1. Introduction

Raw milk cheeses harbor a complex microbiota that develops during processing and contributes to milk acidification, curd formation, and aging, ultimately shaping the sensory characteristics of the final product [1]. However, pathogenic microorganisms can be present in raw milk and/or on contaminated equipment [2]. Several pathogens are recognized as relevant along the dairy chain, including Shiga toxin-producing *Escherichia coli* (STEC), *Listeria monocytogenes*, *Staphylococcus aureus*, and *Salmonella* spp.

*E. coli* comprises a heterogeneous group of Gram-negative bacteria classified into multiple pathotypes [3,4], including the enterohemorrhagic and Shiga toxin-producing groups [3]. STEC strains carry different virulence genes, such as *stx1* and *stx2* for Shiga toxin production, and genes encoding intestinal adhesion factors, such as *eae*, which encodes intimin. In humans, STEC infection most commonly causes diarrhea [3], but in 5–10% of cases it progresses to hemolytic–uremic syndrome (HUS), a serious condition characterized by thrombocytopenia, hemolytic anemia, and acute kidney damage [5,6,7]. This pathogen is particularly concerning because its infectious dose can range from as few as 1 to 700 viable cells [8]. Several risk factors have been identified for progression of STEC infection to HUS, including strain-specific characteristics and virulence factors (e.g., the concurrent presence of both *stx2a* and *eae* genes) [9,10].

Ruminants, particularly cattle, are recognized as the primary reservoir of STEC, shedding the bacteria in feces and contaminating the environment [3]. Human infection is primarily foodborne, arising from animal-derived foods, fruits, vegetables, and drinking water [11]. In 2024, STEC infection was the third most commonly reported foodborne illness in humans in the European Union, with a notification rate of 3.5 cases per 100,000 people [12]. Notably, in 2023, five of the six strong-evidence outbreaks were linked to raw milk products [4]. Since raw milk and raw milk products are important sources of human STEC infection, the adoption of appropriate hygiene practices throughout all stages of production is critical, along with the implementation of control measures across the entire food chain [13].

Under Article 14 of the General Food Law (Reg. (EC) 178/2002) [14], Food Business Operators (FBOs) must not place unsafe products on the market and are responsible for ensuring product safety. In this context, FBOs are expected to implement microbiological own-check plans to verify product safety. Given the low infectious dose of STEC, it is of critical importance to minimize false negative results, including through appropriate sampling strategies. However, the most suitable sampling area within a cheese wheel for STEC detection remains uncertain [15]. Furthermore, to our knowledge, data on STEC behavior in naturally contaminated dairy products remain limited.

European regulations do not provide specific guidelines for STEC sampling strategies to improve detection in dairy products. Reg. (EC) 2073/2005 sets STEC criteria only for sprouts [16]. In Italy, Conferenza Stato-Regioni CSR Resolution n.212 of 10 November 2016 [17] established STEC controls for raw milk cheeses at the distribution stage, setting a limit of “absence in 25 g”.

To close this gap, in March 2022 the Trentingrana Consorzio dei Caseifici Sociali Trentini (CONCAST) established a technical committee together with the Official Laboratory of the Istituto Zooprofilattico Sperimentale delle Venezie (IZSVe) and local veterinary services to manage STEC risk. This collaboration led to the development of the CONCAST guidelines for STEC management, with particular emphasis on sampling procedures. Implemented in January 2023 as a control measure within the framework of good hygiene practices, the guidelines require consortium-affiliated dairies to screen all curd batches intended for the production of raw-milk cheeses aged for up to one year. The CONCAST protocol was subsequently incorporated into the Italian Ministry of Health guidelines on STEC, drafted in 2025 [18]. These national guidelines provide sampling recommendations both for curd during production and for cheeses ready for distribution (recommending sampling from the core).

Several studies using experimentally inoculated raw milk cheeses have reported a reduction in STEC levels during aging, but not complete elimination, with viable cells still isolated after 270 days of maturation [19,20,21,22]. Nonetheless, most of these studies rely on artificially contaminated products, which may not fully reflect naturally occurring contamination during production. Natural contamination may result in uneven STEC distribution within cheese wheels, potentially leading to intermittent detection patterns. Moreover, naturally occurring STEC contamination may remain detectable throughout prolonged cheese aging under real production conditions. Nonetheless, evidence on the behavior of naturally contaminating STEC strains in cheese is limited.

Therefore, this study aimed to provide evidence on the spatial distribution and temporal variability of the detection of naturally contaminating STEC strains in bovine raw milk cheeses, in order to support FBOs’ own-check sampling strategies. First, we evaluated the distribution of STEC within naturally contaminated cheese wheels, with the aim of reducing sampling bias. Second, we investigated changes in STEC detection patterns during the aging of naturally contaminated bovine raw milk washed-rind cheese.

## 2. Materials and Methods

### 2.1. Sample Collection

Between January and February 2023, an intensive sampling plan for STEC risk assessment was implemented at a dairy plant in Trento Province, Northern Italy. All curds (n = 44) produced from bovine raw milk were tested for the presence of STEC. Among the STEC-positive curds (n = 8), three were randomly selected for inclusion in the study. The selected curds were used to produce a washed-rind cheese. All cheese wheels obtained from a single curd were grouped into one batch. The three batches derived from the three positive curds included in the study were designated as A, B, and C. Batch A was used to determine STEC distribution across different areas of the cheese wheel, while batches B and C were used to evaluate STEC behavior throughout a 197–198-day aging period.

### 2.2. Cheese Manufacture

Raw bovine milk was collected from 15 farms in Trento province and delivered daily to the dairy plant. A homemade lactic acid starter culture was added. Cheese was manufactured by rennet coagulation, and the curd was cooked at 46 ± 2 °C for approximately 30 min. Salting consisted of immersion in a 15–20 °Bé brine solution for 2–4 days. Cheese wheels were manufactured by the dairy staff as part of the routine commercial production process. During the first three weeks of ripening, cheese wheels were sponged twice a week with warm diluted brine (1:10 dilution in water) to develop their characteristic aroma. Subsequently, sponging was performed once a week until 107–108 days of ripening. The resulting product was a semi-hard, semi-cooked, washed-rind cheese with a soft, springy, light yellow-white texture with scattered, irregular eyes, sold as semi-aged (60–180 days). Each wheel weighed between 9 and 13 kg and measured approximately 35 cm in diameter and 10 cm in height. Cheese wheels were aged in rooms located in the same dairy plant, at temperatures ranging from 10 °C to 20 °C and relative humidity between 85% and 100%.

### 2.3. Distribution Study: Analysis Within Wheels

**Figure 1:**
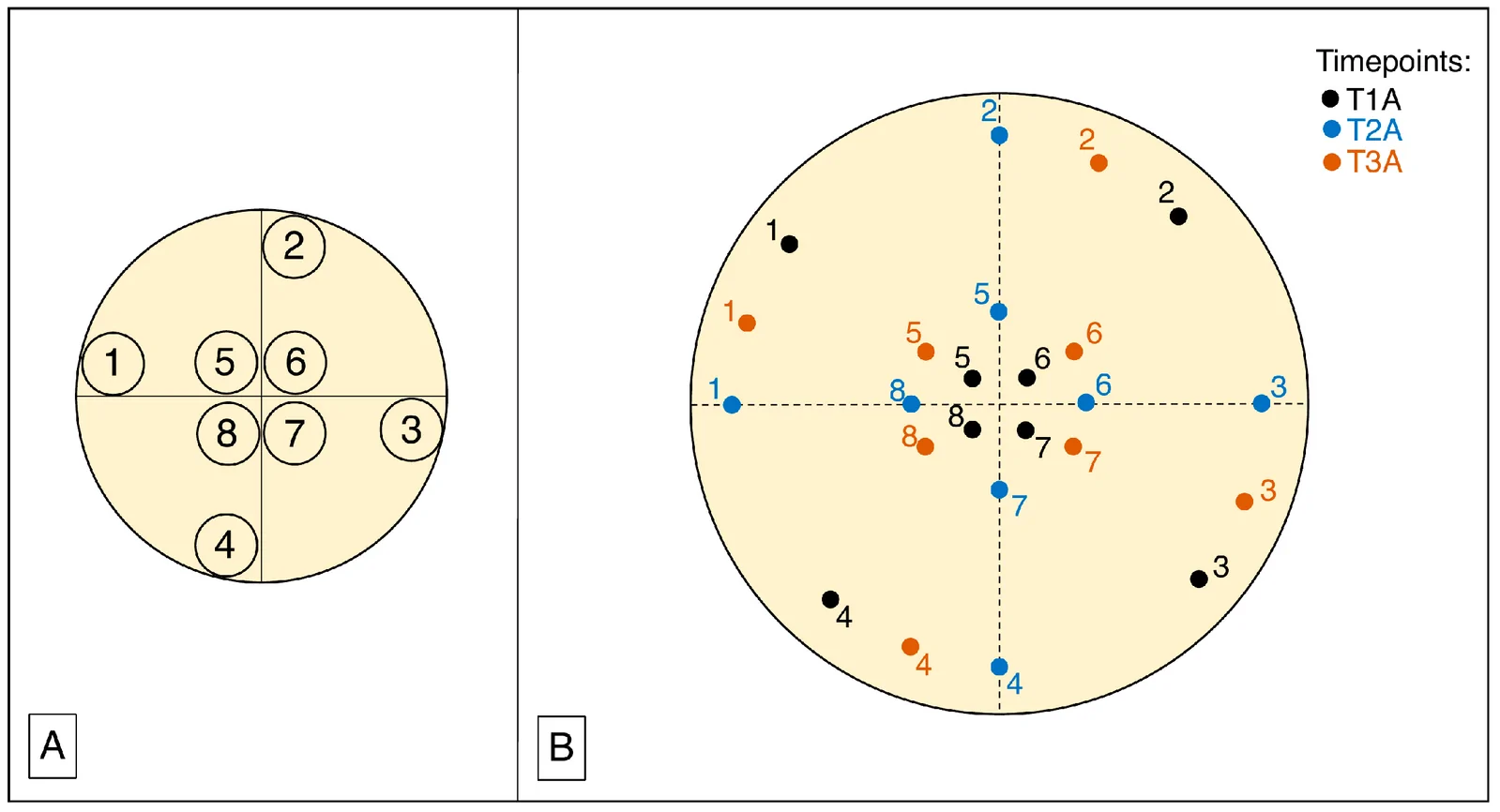
Sampling scheme for cheese wheels belonging to Batch A: (**A**) schematic representation of the eight sampling locations within a cheese wheel; (**B**) exact location of core samples collected at the three sampling times (T1A = 55 days, T2A = 85 days, T3A = 150 days). The external region (sampling points 1, 2, 3, 4) corresponded to the area located within 5 cm of the rind, whereas the core region (sampling points 5, 6, 7, 8) consisted of the central area within a 5 cm radius of the wheel center.

Two cheese wheels from batch A were analyzed. Each wheel was divided into four quarters, each of which was further divided into a core and an external region. Sample locations were identified and numbered from 1 to 8, as shown in Figure 1. The external region was defined as the area within 5 cm of the cheese rind, whereas the core region comprised the central area within a 5 cm radius of the wheel center. Sampling was carried out three times during aging: at 55 days (T1A), 85 days (T2A), and 150 days after production (T3A). At each timepoint, eight samples were taken from the different locations by coring, using a sterile stainless steel trier with an internal diameter of 15 mm. Cheese sampling was conducted in accordance with ISO 707:2008 [23]. Samples of 35–40 g were collected and placed into sterile plastic bags. The holes left in the cheese wheels were filled with melted butter obtained from pasteurized cream, to restore surface integrity and prevent exposure of the cheese interior to the external environment. Butter was chosen because it adheres to the cheese surface and adapts to structural changes occurring in the cheese wheel during aging, reducing the formation of gaps at the sampling site that could facilitate air exchange and potential entry of external contaminants. This procedure was adopted as a physical sealing approach during repeated sampling (according to CONCAST STEC guidelines).

### 2.4. Analysis Within Batches: Wheel Variability and Aging Effect

Fifty cheese wheels from batches B and C (25 wheels from each batch) were analyzed. These batches were produced at the same dairy plant within a 24 h period, using milk collected from the same farms and following the same manufacturing process, with routine cleaning procedures carried out between batches. Analyses were performed at four timepoints during aging: 85–86 days (T1), 121–122 days (T2), 170–171 days (T3), and 197–198 days (T4). Samples were taken from the center of each wheel (to minimize sampling bias) by coring, using a sterile stainless steel trier with an internal diameter of 15 mm. After sampling, the holes were filled with melted butter obtained from pasteurized cream to prevent potential contamination of the cheese interior (according to CONCAST STEC guidelines), and 35–40 g samples were stored in sterile plastic bags.

### 2.5. Microbiological Analysis

**Figure 2:**
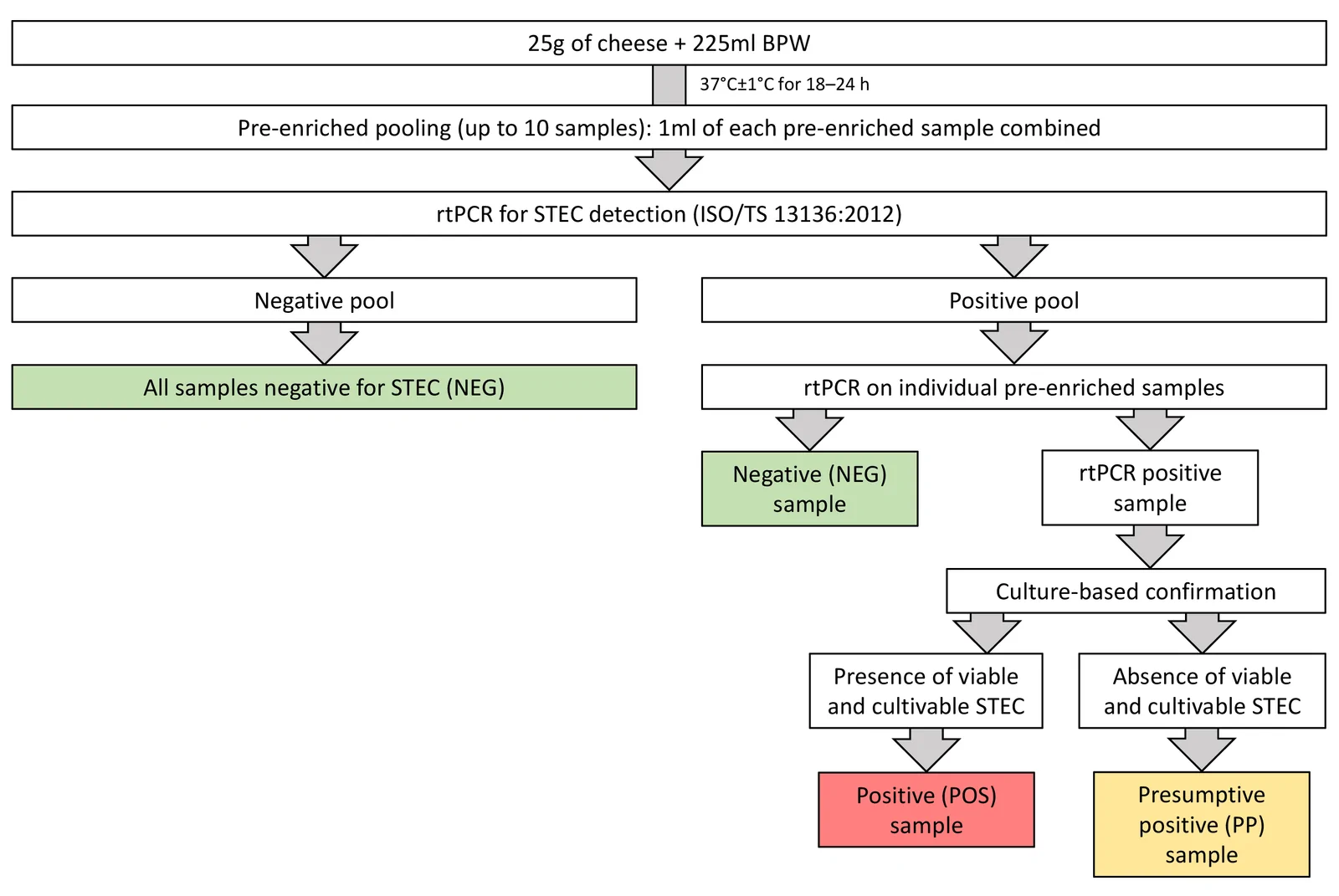
Schematic overview of the analytical workflow adopted for Shiga toxin-producing *Escherichia coli* (STEC) detection and classification of cheese samples into negative (NEG, green boxes), presumptive positive (PP, yellow box), and culture-confirmed positive (POS, red box) according to ISO/TS 13136:2012 [24]. BPW = Buffered Peptone Water; rtPCR = real-time PCR.

Cheese samples were transported to the IZSVe laboratory in Trento Province under refrigerated conditions (5 ± 3 °C) in portable coolers, within 24 h. STEC detection was performed according to ISO/TS 13136:2012 [24], following the pooling method previously described by Weiss et al. [25]. Briefly, 25 g of cheese was pre-enriched with 225 mL of buffered peptone water (BPW, Production Service Centre, IZSVe, Padua, Italy) and incubated at 37 °C ± 1 °C for 18–24 h. Pre-enriched broths were subsequently combined into pools of up to 10 samples for DNA extraction and real-time PCR (rtPCR) screening targeting the *stx1*, *stx2*, and *eae* genes [24]. The limit of detection (LOD) of the molecular screening method was 5 cfu/sample, according to ISO 6887-1:2017 [26] and ISO 16140-4:2020 [27]. Pools testing negative for both *stx1* and *stx2* genes were classified as negative (NEG), and all individual samples belonging to those pools were consequently considered negative. For pools testing positive for *stx1* and/or *stx2*, DNA extraction and rtPCR analysis were repeated individually on each corresponding pre-enriched broth. Samples testing positive by rtPCR for *stx1* and/or *stx2* genes were subsequently subjected to culture isolation. A sample was classified as positive (POS) if viable and cultivable STEC were isolated, or as presumptive positive (PP) if no cultivable STEC could be recovered. STEC isolation was performed on MacConkey agar (McC, Biolife Italiana s.r.l., Milan, Italy), Tryptone Bile X-Glucuronide agar (TBX, Biokar Diagnostics, Allonne, France), and Sorbitol McC with cefixime tellurite (CT-SMAC, Biolife Italiana s.r.l., Milan, Italy). The LOD of the culture-based confirmation method was 5 cfu/sample. A flowchart summarizing the STEC detection and confirmation procedure is presented in Figure 2.

All rtPCR-positive pre-enriched samples were further screened for the five major STEC serogroups of public health concern in Europe (top-5: O157, O111, O26, O103, O145), according to ISO/TS 13136:2012 [24], and for serotype O104:H4 using the ISS EU-RL VTEC method 04 [28].

Water activity (aw) was measured according to ISO 18787:2017, using a WaterLab (Steroglass, Perugia, Italy) instrument [29]. pH was determined according to MFHPB-03:2014, using a SensION+ PH31 pH meter (HACH Lange S.r.l., Lainate, MI, Italy) [30].

### 2.6. Statistical Analysis

For statistical purposes, POS and PP samples were considered together. All statistical analyses were performed using R software (v. 4.5.2) [31]. STEC results were expressed as prevalence, with 95% exact binomial confidence intervals. Differences in STEC prevalence between timepoints within the same batch were assessed using McNemar’s test, while differences between batches at the same timepoint were evaluated using the two-sample test for proportions (prop.test function). A *p*-value < 0.05 was considered statistically significant.

## 3. Results

### 3.1. Distribution Study: Analysis Within Wheels

Batch A cheese was used for the distribution study. Eight samples were taken from each wheel at the three timepoints. STEC detection in each wheel, by sampling location (external area or core) and timepoint, is summarized in Table 1. All positive or presumptive positive samples carried the *stx2* gene, and all tested negative for *stx1* and *eae* genes by rtPCR. Neither the top-5 serogroups nor serotype O104:H4 were detected.

The two wheels produced from the STEC-positive curd showed STEC detection prevalence values of 87.5% (wheel 1) and 62.5% (wheel 2) after 55 days of aging. External regions showed higher detection rates than the core regions (100.0% vs. 75.0% in wheel 1, 75.0% vs. 50.0% in wheel 2). At T2A, external regions again showed higher detection rates than the core (100.0% vs. 50.0% in wheel 1; 75.0% vs. 50.0% in wheel 2). At T3A, only one sample from wheel 1 tested positive (12.5%), while wheel 2 showed a detection rate consistent with T1A and T2A (62.5%). In wheel 1, the positive sample at T3A came from the external region, whereas the core yielded no positive results. By contrast, the external region of wheel 2 showed a lower detection rate than the core (50.0% vs. 75.0%).

**Figure 3:**
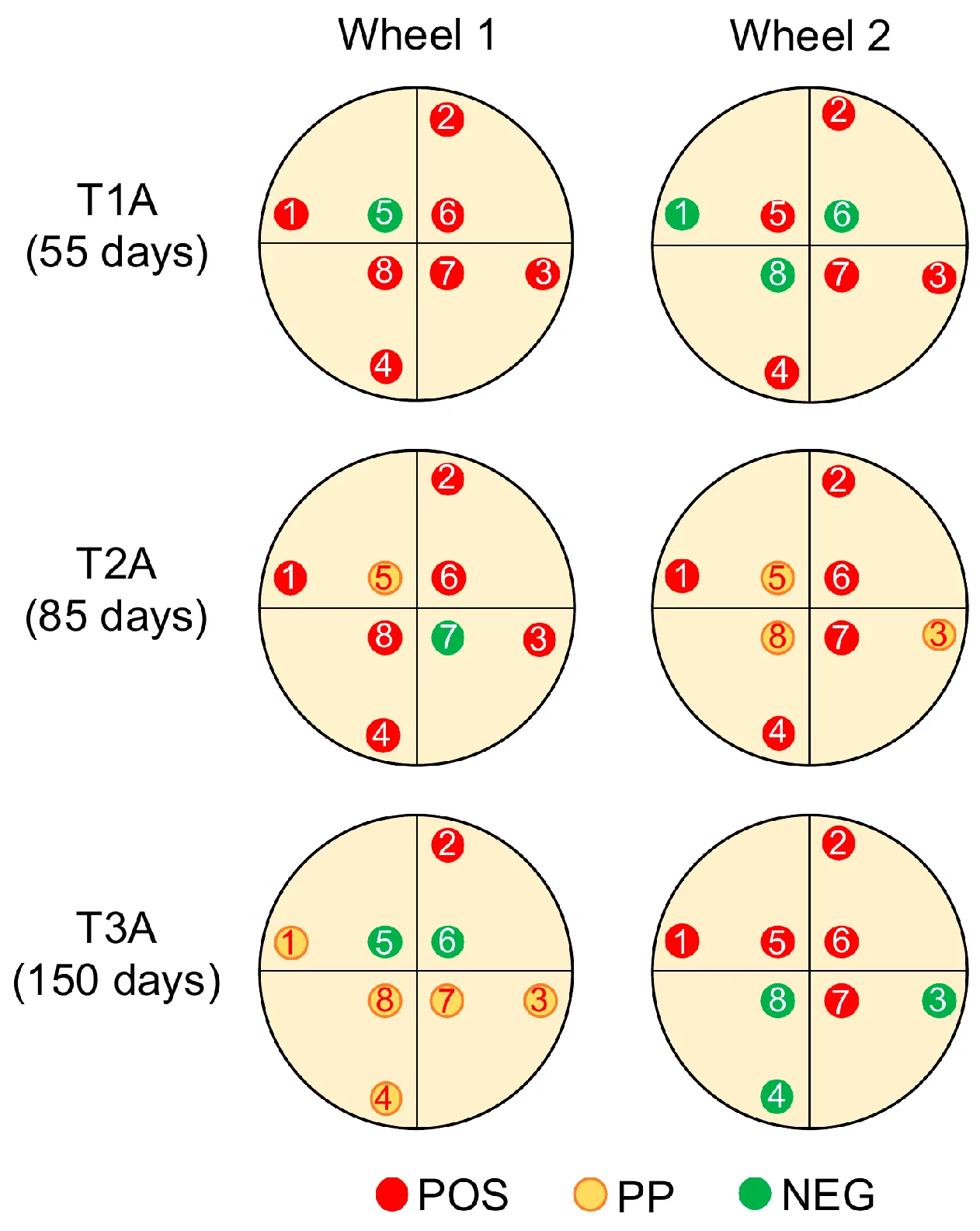
Distribution of Shiga toxin-producing *Escherichia coli* (STEC; ISO/TS 13136:2012 [24]) and/or toxin genes in two cheese wheels from batch A at three different aging durations (T1A = 55 days, T2A = 85 days, T3A = 150 days). POS = positive sample, positive by rtPCR for *stx1* and/or *stx2* genes and culture-positive. PP = presumptive positive sample, positive by rtPCR for *stx1* and/or *stx2* genes but culture-negative. NEG = negative sample, negative by rtPCR for *stx1* and *stx2* genes.

The distribution of positive and negative samples is shown in Figure 3.

### 3.2. Analysis Within Batches: Wheel Variability and Aging Effect

The second part of the study focused on the detection of STEC over time in cheese batches B and C, each derived from a naturally contaminated curd. For batch B, 25 wheels were analyzed at four aging timepoints, for a total of 100 analyses. For batch C, 25 wheels were analyzed at the first timepoint and 24 wheels at each subsequent sampling time, as the 25th wheel was excluded from the study for technical reasons.

Aw and pH were determined at each aging timepoint using one wheel from each batch; results are shown in Table 2.

STEC detection results and the genes identified by rtPCR in batch B are summarized in Table 3. Most positive or presumptive positive samples carried the *stx2* gene, whereas *stx1* was detected only in three presumptive positive samples. The *eae* gene was amplified in only one sample, although no bacterium carrying this gene could be isolated. Neither the top-5 serogroups nor the O104:H4 serotype was detected.

Nine batch B cheese wheels tested negative at all timepoints. The remaining 16 wheels showed at least one positive or presumptive positive result during the study, with positive/presumptive positive and negative results alternating throughout the sampling period. Four wheels tested negative at the first timepoint but subsequently tested positive at later timepoints (POS result at T2 and/or T4). Six other wheels were negative at T1 but presumptive positive at later timepoints (PP result at T3 and T4). By the end of the study (T4), seven wheels that had been positive or presumptive positive at earlier timepoints tested negative.

Among Batch B cheese wheels, no significant differences in STEC prevalence were observed across aging timepoints, except between T2 and T4 (*p* = 0.0455), where the detection of positive/presumptive positive samples was significantly higher at T4 (POS and PP results were considered together in the statistical analysis).

Results for batch C at the four aging timepoints (T1, T2, T3, T4) are summarized in Table 4. All positive or presumptive positive samples carried the *stx2* gene. No samples tested rtPCR-positive for *stx1* or *eae*, and neither the top-5 serogroups nor serotype O104:H4 was detected.

No batch C cheese wheel tested negative at all timepoints. Six wheels tested positive at every timepoint, while six wheels that were presumptive positive at T1 became positive at subsequent timepoints. At T1, STEC was not detected in four wheels that later tested positive (POS result). By the end of the study (T4), only 2/24 (8.33%) batch C cheese wheels tested negative.

When comparing timepoints, T4 showed a significantly higher detection of positive/presumptive positive samples than T1 (*p* = 0.0348).

When comparing the same aging timepoints between the two batches, the estimated prevalence of positive/presumptive positive samples was significantly higher for batch C than for batch B at every timepoint (*p* = 0.0016, *p* < 0.0001, *p* = 0.0020, and *p* < 0.0001 for T1, T2, T3, and T4, respectively).

## 4. Discussion

The present study investigated the distribution of STEC in naturally contaminated washed-rind cheese produced from STEC-positive curds, as well as its detection patterns during aging. FBOs are responsible for ensuring the safety of their products, and in the dairy sector this includes implementing own-check plans for STEC monitoring. Our findings highlight the challenges of reliably detecting STEC in washed-rind raw milk cheese, showing that its heterogeneous distribution within cheese wheels, together with its intermittent detection over time, can compromise sampling effectiveness.

STEC-positive samples were detected in both the external and core regions of cheese wheels at all sampling times. At the first two timepoints, external areas showed higher STEC prevalence. According to Ercolini et al., who modeled STEC distribution in Grana Padano cheese, the external rind of the curd is more likely to support STEC survival than the core [32]. Conversely, Miszczycha and colleagues reported a smaller STEC decrease in the core than in the rind during the ripening of uncooked pressed cheese [20]. Similarly, after 150 days of aging, wheel 2 showed higher STEC prevalence in the core than in the external area. Only two wheels from the selected curd were available for this detailed, destructive sampling approach, because these batches were produced within a commercial framework. The experiment was therefore designed as an exploratory spatial investigation rather than a prevalence study. Although the limited number of observations limits the generalizability of these results, they nonetheless point to an uneven distribution of STEC within naturally contaminated washed-rind cheeses of 35 cm diameter, produced under standard processing conditions.

Studies using inoculated pathogens have shown that the distribution of contamination between rind and core depends on both the STEC strain and the cheese type [32,33]. Moreover, sampling locations that tested negative at the first timepoint later tested positive at T2A and T3A. This variability complicates the identification of an optimal aging duration and sampling location for detecting STEC contamination. Since pathogen concentrations are expected to be higher in the curd [33,34], sampling at an earlier production stage may improve STEC detection. However, this study did not evaluate the relative sensitivity of STEC detection in curd versus cheese. Further research is therefore needed to directly compare detection sensitivity between curd and finished cheese, as well as to assess whether curd sampling may improve detection. Larger studies across different cheese types are also needed to further investigate the inhomogeneous distribution of STEC within wheels.

The time-course analysis of naturally contaminated washed-rind cheese wheels from two different batches (each produced from a positive curd) showed that 198 days of aging were not sufficient to guarantee the absence of STEC strains: a substantial proportion of wheels tested STEC-positive at the end of the study, particularly in batch C, where the prevalence of positive/presumptive positive samples was significantly higher at T4 than at earlier sampling times. Since we did not quantify STEC concentrations or genotype STEC strains, these findings should not be taken as evidence of bacterial growth during aging; they do indicate, however, that prolonged ripening cannot guarantee the absence of STEC from naturally contaminated washed-rind cheese.

Both batches underwent only minor physicochemical changes during ripening, with a slight increase in pH and a limited reduction in water activity. These variations are consistent with the characteristics of washed-rind cheese and are unlikely to have caused substantial STEC inactivation [33].

Several studies have described the persistence of artificially inoculated STEC strains for up to 270 days [19,20,21,22], with a gradual decrease in concentration during aging [20,35,36]. Since we did not genotype the STEC strains contaminating the cheese, we cannot determine whether STEC detected at different timepoints stemmed from persistence of the initial contamination or from subsequent contamination events, nor whether the same or different strains were involved. Nonetheless, we observed higher proportions of STEC-positive/presumptive positive samples at the last stage of aging, mainly in batch C. This discrepancy with the literature may also reflect variability in the behavior of different STEC strains, or the influence of environmental factors such as the physicochemical characteristics of the cheese, the production technology, and the composition of the resident microbial community, including lactic acid bacteria [20,37,38]. Moreover, field STEC strains can behave differently from laboratory strains in the same matrix [39]. Alternatively, STEC detected at later timepoints may have originated from environmental contamination during cheese manipulation (e.g., coring or sponging of the rind with brine) [34]. Coring was performed with a sterile trier to minimize the risk of cross-contamination between sampling points [23], whereas repeated sponging with brine represents a potential route for external contamination throughout aging, since it involves the entire rind surface and is repeated over time during the first three months. However, the microbial community harbored on the rind of washed-rind cheeses plays a protective role against undesirable bacteria, including STEC [40]. Sampling was performed in the core region of the wheel to limit external-contamination issues, but further studies are needed to assess whether STEC can penetrate a cheese wheel following superficial contamination. Finally, brine sponging was performed only during the first three months of aging and was discontinued before T2 sampling. In batch B, culture-confirmed STEC-positive samples were lowest at T2 (4.0%, the end of the sponging period), then increased at later sampling times (12.0% at T3 and 20.0% at T4). Similarly, batch C showed fluctuations in STEC detection prevalence over time rather than a progressive increase. These patterns do not suggest a clear temporal association between brine sponging and STEC detection and are compatible with a heterogeneous STEC distribution within cheese wheels. However, subsequent external contamination events during aging cannot be ruled out entirely and should be considered as a potential food safety concern by FBOs, highlighting the importance of maintaining proper hygienic practices throughout manufacturing [41].

A substantial proportion of samples yielded presumptive positive results (10.0% and 16.5% across the study period for batches B and C, respectively), for which the presence of viable *E. coli* cells carrying the *stx1* and/or *stx2* genes could not be confirmed. Several factors could explain this: the samples may not have contained viable STEC cells, the DNA targets for *stx1* and/or *stx2* may have originated from free phages in the matrix [42], or the pathogen may have been present at low concentrations, below the limit of detection [43]. From a microbiological standpoint, presumptive positive results cannot be considered equivalent to confirmed STEC contamination. From a food safety perspective, however, they should not be disregarded. Under EU Regulation 178/2002, FBOs must not place unsafe food products on the market, and Article 14 defines a food as unsafe if “it is considered to be injurious to health or unfit for human consumption” [14]. Presumptive positive results therefore warrant careful consideration, as they may signal STEC circulation within the production plant [42,44]. Within this framework, the Italian guidelines on STEC control give the FBO the option of adopting a cautious approach, treating such results as positive in order to increase vigilance and reduce the possibility of marketing at-risk products [18]. In line with this, presumptive positive samples were grouped with culture-confirmed positive samples for the statistical analysis performed in the present study. This conservative approach reflects a “worst-case scenario”, in which PCR-positive/culture-negative samples could potentially correspond to viable STEC present at levels below the limit of detection, rather than a true absence of the pathogen. This scenario needs to be taken into consideration, given that STEC can cause disease with as little as one viable cell [8,45]. Notably, wheels that tested presumptive positive at T1 later tested positive. While this cannot confirm that viable STEC were present in the initial samples, and subsequent external contamination cannot be ruled out, it supports the hypothesis that at least some presumptive positive results may reflect low levels of STEC contamination close to the detection threshold.

Positive and presumptive positive samples (considered jointly) were significantly more frequent in batch C than in batch B at every timepoint. Since both batches were produced on consecutive days from milk collected at the same farms and processed under the same manufacturing protocol, this difference may reflect variation in the initial level and/or spatial distribution of STEC contamination within the curd. This variation in contamination burden across successive days is consistent with the intermittent shedding of STEC in ruminant feces [41] and with variation in hygiene practices during milking [46]. Monitoring STEC presence and farm conditions through appropriate surveillance programs, together with effective cleaning and disinfection of all equipment, would therefore help reduce the likelihood of bacterial contamination along the entire raw milk cheese supply chain, thereby protecting food safety and consumer health [13,32,47].

Overall, our results highlight an uneven distribution of field STEC strains, both within and between wheels of washed-rind raw milk cheese, along with variation in prevalence between batches. These patterns may reflect uneven STEC contamination of the raw milk itself, a non-homogeneous distribution of STEC within individual wheels, or subsequent contamination events during aging. Together, these aspects of STEC behavior contribute to the difficulty of designing an effective sampling plan to verify product safety for consumer protection. The FBO must define, on a statistical basis, the number of wheels to be tested within a batch according to the desired confidence level, production volume, and expected prevalence [18]. Nevertheless, as observed in both batches B and C, a single sample from a given wheel may test negative even though the same wheel later tests positive. This suggests a high risk of false negative results when relying on a single sample, and further supports the hypothesis of a heterogeneous distribution of STEC within the wheel. FBOs should carefully consider which stage of production offers the most reliable point for sampling. According to the literature, the curd is likely to be the more sensitive matrix, since it concentrates bacteria potentially present in whole milk and may therefore contain higher STEC levels than the resulting cheese, facilitating detection [34]. Early identification of a positive curd also allows the FBO to become aware of positive cheese batches as early as the start of production, and to plan the most appropriate measures for the cheese during aging and at the end of production, in order to contain the risk posed by STEC. Although this study did not directly compare curd and cheese sampling, our findings provide additional support for sampling at earlier stages of production. These procedures should be accompanied at all times by strict hygienic and management practices, in order to minimize the risk of external contamination of cheese during aging [18,41]. Further studies on other raw milk cheese varieties are needed to confirm whether these observations extend beyond the cheese examined here.

Unlike previous studies, which artificially contaminated the cheese products with laboratory STEC strains, we evaluated field strains naturally present in raw milk and monitored their presence throughout the production process. We chose to evaluate the detection patterns of field strains to give the FBO an indication of how STEC strains detected in the dairy facility may behave, highlighting the challenges that may be encountered during cheese sampling for STEC isolation. Although we did not further characterize the isolated strains, none belonged to the European top-5 serogroups. However, recent studies suggest that serogroups and the presence of the *eae* gene are weak predictors of pathogenicity and clinical outcomes [48]. Nevertheless, it should be noted that all isolated strains carried the *stx2* gene, which is believed to be the toxin most involved in severe cases of human disease [9,49]. Moreover, this gene is located on the genome of a bacteriophage, and can therefore be horizontally transferred to other bacterial strains, which could thereby become disease agents [50,51]. It is therefore more urgent for the FBO to detect the presence of these genes in viable bacteria than to determine their specific serogroup. However, strain typing would be valuable for assessing the co-occurrence of different strains and their dynamics over time.

A limitation of the study is that STEC contamination could not be quantified at either the start of cheese-making or during aging. This reflects a technical limitation, since no reliable methods currently allow such quantification in non-experimental settings. Although coliform and generic *E. coli* counts may give an indirect, overall indication of *E. coli* contamination, they cannot be used to specifically quantify STEC; moreover, no selective culture medium has been validated for the quantitative enumeration of STEC in cheese matrices. Our results therefore describe the occurrence and detection of STEC rather than changes in bacterial concentration during aging. Molecular typing was also not performed, which prevented assessment of whether one or multiple STEC strains were present in the cheese wheels and limited the interpretation of strain persistence over time. Finally, the small number of samples collected from batch A limited our ability to statistically compare STEC presence between the core and external regions. Since all analyses were carried out on a single cheese variety from a single dairy plant, these findings should be extended to other cheese types or production systems with caution.

Further studies should examine larger numbers of naturally contaminated cheeses produced across different production technologies and facilities, combining microbiological isolation with strain typing. A more detailed understanding of how STEC interacts with biotic (e.g., lactic acid bacteria) and abiotic factors (e.g., temperature, pH, aw, organic acid concentration) during production would further clarify how naturally contaminating STEC persists in raw milk cheese [38].

Overall, this study assesses the detection patterns of field STEC strains in naturally contaminated batches of washed-rind raw milk cheese under commercial processing conditions. By focusing on the practical difficulties of STEC sampling and detection, our findings aim to help FBOs better understand the challenges of STEC monitoring and identify the most appropriate measures for ensuring safe cheese production.

## 5. Conclusions

This study showed that the distribution of naturally occurring STEC within batches of washed-rind raw milk cheese is inhomogeneous, highlighting the difficulties involved in STEC detection in the final product. Similar variability was observed within individual cheese wheels, suggesting that detection differences might stem from uneven bacterial distribution. This variability suggests that sampling should target the curd, as it may represent the production stage at which STEC is most likely to be detected. Additionally, STEC was detected in cheese wheels up to day 198 of aging, indicating that contamination may remain detectable beyond medium-to-long aging periods. This underscores the need to assess STEC survival on cheese-type-specific basis, as production methods and aging time may affect contamination levels differently. Moreover, it underscores the importance of consistently applying good hygienic practices throughout the production process to reduce the risk of cheese contamination during aging. The purpose of this study was not to provide specific evidence on STEC persistence in cheese or to characterize circulating bacterial strains. Rather, our findings highlight the heterogeneous detection patterns of naturally contaminating field strains under commercial production conditions, underscoring the challenges of designing an effective sampling plan, as well as the need to update current European regulations and to assess STEC pathogenicity more broadly, beyond the current focus on a limited number of serogroups. It is therefore crucial for FBOs to implement systematic monitoring throughout the food chain to reduce STEC risk in cheese production.

## Figures and Tables

**Table 1:** Detection of Shiga toxin-producing *Escherichia coli* (STEC; ISO/TS 13136:2012 [24]) at different aging timepoints in two cheese wheels belonging to batch A. Aging timepoints: T1A = 55 days, T2A = 85 days, T3A = 150 days. POS = positive sample, positive by rtPCR for *stx1* and/or *stx2* genes and culture-positive. PP = presumptive positive sample, positive by rtPCR for *stx1* and/or *stx2* genes but culture-negative. NEG = negative sample, negative by rtPCR for *stx1* and *stx2* genes. CI_95_ = 95% Confidence Interval.

		T1A		T2A		T3A	
Sample Location	Region	Wheel 1	Wheel 2	Wheel 1	Wheel 2	Wheel 1	Wheel 2
1	External	POS	NEG	POS	POS	PP	POS
2	External	POS	POS	POS	POS	POS	POS
3	External	POS	POS	POS	PP	PP	NEG
4	External	POS	POS	POS	POS	PP	NEG
% POS samples	External	100.0% CI_95_ [39.76;100.00]	75.0% CI_95_ [19.41;99.37]	100.0% CI_95_ [39.76;100.00]	75.0% CI_95_ [19.41;99.37]	25% CI_95_ [0.63;80.6]	50% CI_95_ [6.76;93.24]
5	Core	NEG	POS	PP	PP	NEG	POS
6	Core	POS	NEG	POS	POS	NEG	POS
7	Core	POS	POS	NEG	POS	PP	POS
8	Core	POS	NEG	POS	PP	PP	NEG
% POS samples	Core	75.0% CI_95_ [19.41;99.37]	50.0% CI_95_ [6.76;93.24]	50.0% CI_95_ [6.76;93.24]	50.0% CI_95_ [6.76;93.24]	0.0% CI_95_ [0.00;60.24]	75.0% CI_95_ [19.41;99.37]
% POS samples	Whole wheel	87.5% CI_95_ [47.35;99.68]	62.5% CI_95_ [24.49;91.48]	75.0% CI_95_ [34.91;96.81]	62.5% CI_95_ [24.49;91.48]	12.5% CI_95_ [0.32;52.65]	62.5% CI_95_ [24.49;91.48]

**Table 2:** Water activity (aw) and pH of cheese batches B and C at different aging timepoints (T1 = 85–86 days, T2 = 121–122 days, T3 = 170–171 days, T4 = 197–198 days).

	Batch B		Batch C	
Aging Duration	aw	pH	aw	pH
T1	0.97	5.5	0.97	5.5
T2	0.97	5.5	0.97	5.7
T3	0.97	5.6	0.97	5.7
T4	0.96	5.7	0.96	5.8

**Table 3:** Detection of Shiga toxin-producing *Escherichia coli* (STEC; ISO/TS 13136:2012 [24]) and STEC-associated genes at different aging timepoints in 25 cheese wheels belonging to batch B. Aging timepoints: T1 = 85–86 days, T2 = 121–122 days, T3 = 170–171 days, T4 = 197–198 days. POS = positive sample, positive by rtPCR for *stx1* and/or *stx2* genes and culture-positive. PP = presumptive positive sample, positive by rtPCR for *stx1* and/or *stx2* genes but culture-negative. NEG = negative sample, negative by rtPCR for *stx1* and *stx2* genes. CI_95_ = 95% Confidence Interval.

Cheese Wheel Number	T1		T2		T3		T4	
	Result	Positive Gene	Result	Positive Gene	Result	Positive Gene	Result	Positive Gene
B1	PP	*stx1, stx2*	NEG	-	PP	*stx1*	POS	*stx2*
B2	PP	*stx2*	NEG	-	POS	*stx2*	NEG	-
B3	NEG	-	NEG	-	NEG	-	NEG	-
B4	NEG	-	NEG	-	NEG	-	POS	*stx2*
B5	POS	*stx2*	NEG	-	POS	*stx2*	PP	*stx2*
B6	NEG	-	NEG	-	NEG	-	NEG	-
B7	NEG	-	NEG	-	NEG	-	NEG	-
B8	POS	*stx2*	NEG	-	POS	*stx2*	NEG	-
B9	NEG	-	NEG	-	PP	*stx2*	NEG	-
B10	POS	*stx2*	NEG	-	NEG	-	NEG	-
B11	NEG	-	NEG	-	NEG	-	NEG	-
B12	POS	*stx2*	NEG	-	NEG	-	NEG	-
B13	NEG	-	NEG	-	NEG	-	NEG	-
B14	NEG	-	NEG	-	NEG	-	POS	*stx2*
B15	NEG	-	NEG	-	NEG	-	NEG	-
B16	NEG	-	NEG	-	PP	*stx2*	NEG	-
B17	NEG	-	NEG	-	NEG	-	POS	*stx2*
B18	NEG	-	NEG	-	NEG	-	PP	*stx2*
B19	NEG	-	NEG	-	NEG	-	NEG	-
B20	NEG	-	NEG	-	NEG	-	PP	*stx1*
B21	NEG	-	NEG	-	NEG	-	NEG	-
B22	NEG	-	NEG	-	NEG	-	PP	*stx2*
B23	NEG	-	NEG	-	NEG	-	NEG	-
B24	NEG	-	NEG	-	PP	*stx2, eae*	NEG	-
B25	NEG	-	POS	*stx2*	NEG	-	POS	*stx2*
% POS samples	16.0% CI_95_ [4.54;36.08]		4.0% CI_95_ [0.10;20.35]		12.0% CI_95_ [0.98;26.03]		20.0% CI_95_ [6.83;40.70]	
% PP samples	8.0% CI_95_ [0.98;26.03]		0.0% CI_95_ [0.00;13.72]		16.0% CI_95_ [4.54;36.08]		16.0% CI_95_ [4.54;36.08]	

**Table 4:** Detection of Shiga toxin-producing *Escherichia coli* (STEC; ISO/TS 13136:2012 [24]) at different aging timepoints in 25 cheese wheels belonging to batch C. C25 was not analyzed after T1 due to technical reasons. Aging timepoints: T1 = 85–86 days, T2 = 121–122 days, T3 = 170–171 days, T4 = 197–198 days. POS = positive sample, positive by rtPCR for *stx1* and/or *stx2* genes and culture-positive. PP = presumptive positive sample, positive by rtPCR for *stx1* and/or *stx2* genes but culture-negative. NEG = negative sample, negative by rtPCR for *stx1* and *stx2* genes. CI_95_ = 95% Confidence Interval.

Cheese Wheel Number	T1	T2	T3	T4
C1	POS	POS	NEG	POS
C2	PP	POS	PP	POS
C3	POS	NEG	POS	POS
C4	NEG	POS	POS	POS
C5	NEG	POS	POS	POS
C6	POS	NEG	PP	POS
C7	PP	POS	NEG	POS
C8	PP	POS	PP	NEG
C9	PP	POS	POS	POS
C10	POS	POS	POS	POS
C11	POS	POS	POS	POS
C12	NEG	NEG	NEG	POS
C13	POS	POS	POS	POS
C14	PP	NEG	PP	POS
C15	NEG	POS	POS	POS
C16	POS	POS	PP	POS
C17	PP	NEG	PP	POS
C18	POS	NEG	NEG	POS
C19	POS	POS	PP	NEG
C20	POS	POS	POS	POS
C21	POS	NEG	POS	PP
C22	POS	POS	POS	POS
C23	POS	POS	PP	POS
C24	POS	POS	POS	POS
C25	PP	-	-	-
% POS samples	56.0% CI_95_ [34.93;75.60]	70.8% CI_95_ [48.90;87.38]	50.0% CI_95_ [29.12;70.87]	87.5% CI_95_ [67.64;97.34]
% PP samples	28.0% CI_95_ [12.07;49.39]	0.0% CI_95_ [0.00;14.25]	33.3% CI_95_ [15.63;55.32]	4.2% CI_95_ [0.10;21.12]

## Data Availability

The original contributions presented in this study are included in the article. Further inquiries can be directed to the corresponding author.

## Abbreviations

The following abbreviations are used in this manuscript:

CONCAST	Consorzio dei Caseifici Sociali Trentini
ECDC	European Centre for Disease Prevention and Control
EFSA	European Food Safety Authority
FBO	Food Business Operator
HUS	Hemolytic–Uremic Syndrome
IZSVe	Istituto Zooprofilattico Sperimentale delle Venezie
LOD	Limit of Detection
NEG	Negative for *E. coli* STEC
POS	Positive for *E. coli* STEC
PP	Presumptive positive for *E. coli* STEC
rtPCR	Real-Time PCR
STEC	Shiga toxin-producing *Escherichia coli*
